# A case report of cutaneous *Brugiapahangi* infection presenting with recurrent lymphangitis and a subcutaneous nodule

**DOI:** 10.1016/j.jdcr.2026.04.017

**Published:** 2026-04-20

**Authors:** Tanapong Wongrat, Panitta Sitthinamsuwan, Patsharaporn T. Sarasombath, Pattriya Jirawattanadon, Chanisada Wongpraparut

**Affiliations:** a Faculty of Medicine Siriraj Hospital, Department of Dermatology, Mahidol University, Bangkok, Thailand; b Faculty of Medicine Siriraj Hospital, Department of Pathology, Mahidol University, Bangkok, Thailand; c Siriraj Integrative Center for Neglected Parasitic Diseases, Faculty of Medicine Siriraj Hospital, Department of Parasitology, Mahidol University, Bangkok, Thailand

**Keywords:** *Brugia pahangi*, cutaneous filariasis, lymphangitis, subcutaneous nodule, zoonotic infection

## Introduction

Lymphatic filariasis remains a major neglected tropical disease with a substantial global burden. The World Health Organization estimates that more than 51 million people are infected, and 657 million people are living in endemic areas.^1^ Most infections are caused by *Wuchereria bancrofti* or *Brugia malayi*, but human infections with zoonotic filariae are increasingly reported, raising concern about their potential for emergence. Dermatologic presentations vary depending on parasite localization: human lymphatic filariasis classically causes chronic lymphedema, whereas zoonotic filariae may migrate through subcutaneous tissues and mimic other helminthic infections.^2^ We describe a case of subcutaneous human *Brugia pahangi* infection with an unusual presentation that posed significant diagnostic challenges and underscores its potential as an emerging zoonosis.

## Case report

**Fig 1:**
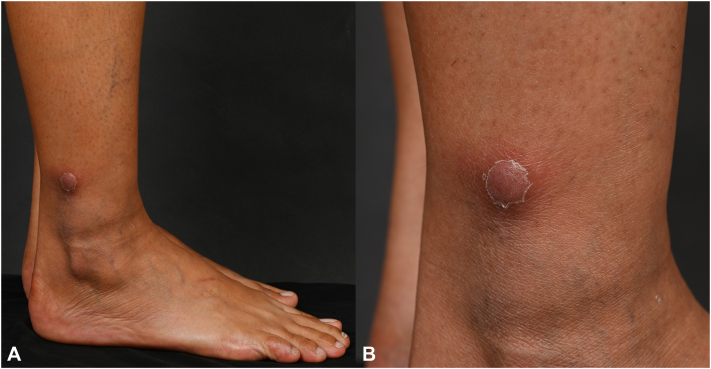
**A** and **B,** a solitary erythematous subcutaneous nodule, measuring 1 cm in diameter on the right lateral ankle.

A 49-year-old woman from southern Thailand presented with a 6-month history of recurrent right-leg lymphangitis, occasionally accompanied by painful right inguinal lymphadenopathy. A biopsy from the affected node showed reactive lymphoid hyperplasia. She received antibiotics for presumed bacterial lymphangitis with temporary resolution. Four months later, she developed a new painful erythematous nodule on her right leg. The patient was previously healthy and denied fever, malaise, systemic symptoms, recent travel, or exposure to animals or undercooked meat. Examination revealed a solitary 1-cm erythematous dermal-subcutaneous nodule over the right lateral ankle without other lesions or lymphadenopathy (Fig 1, *A* and *B*).

**Fig 2:**
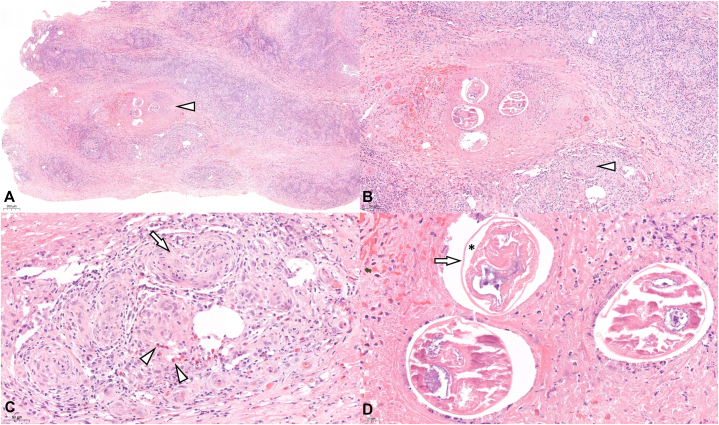
**A,** Predominant lobular panniculitis with mixed inflammation, and transverse sections of filarial nematode (*arrowhead*) (hematoxylin and eosin, original magnification ×40); **B,** Transverse section of degenerative filarial nematode with granulomatous inflammation (*arrowhead*) (hematoxylin and eosin, original magnification ×100) **C,** Some granulomatous inflammation (*arrow*) with scattered eosinophils (*arrowheads*) identified (hematoxylin and eosin, original magnification ×200); **D,** Transverse section of degenerative filarial nematode with pseudocoelom (*asterisk*), a characteristic feature of nematodes and smooth thin cuticle (*arrow*) (hematoxylin and eosin, original magnification ×400).

Skin biopsy revealed lobular panniculitis with mixed neutrophil and eosinophil infiltration and focal granulomatous inflammation. Degenerated cross sectioned nematodes were noted among the inflammation (Fig 2, *A*-*D*). As the parasite was markedly degenerated, species identification based solely on histopathology was limited. Given the patient’s migratory cutaneous lesions and the endemicity of both filarial nematodes and *Gnathostoma* in Thailand, serologic testing for filarial IgG4 and *Gnathostoma* antibodies was performed. Filarial IgG4 was negative, whereas *Gnathostoma* serology was positive; however, the nematode displayed a smooth cuticle, which is inconsistent with the cuticular spines characteristic of *Gnathostoma*, making this serologic result unlikely to represent true infection. Diethylcarbamazine provocation test or night blood examination for microfilaremia was not performed in this patient.

Therefore, molecular identification was pursued using PCR on DNA extracted from paraffin-embedded tissue. PCR amplification of the cytochrome c oxidase subunit I gene and the mitochondrial 12S rRNA gene of filarial nematodes was performed, followed by sequencing of the resulting amplicons. The cytochrome c oxidase subunit I and 12S rRNA sequences of the causative species are 99.7% and 100% identical to *Brugia pahangi*, GenBank accession numbers MK250710.1 and AM779851.1, respectively. The sequences were submitted to NCBI and are available under the accession numbers PX518915 and PX518916.

The patient received 2 daily doses of ivermectin (12 mg/dose). At 2 weeks, the surgical site healed well with no new lesions. By 3 months, the lesion had completely resolved, leaving only a surgical scar.

## Discussion

*B* *pahangi* is endemic in South and Southeast Asia and has been reported in domestic animals such as dogs and cats.^3^^,^^4^ Several mosquito genera, including *Armigeres*, *Mansonia*, and *Aedes*, have been implicated as potential vectors, however, *Armigeres subalbatus* is the most well-established natural vector reported in endemic regions.^5^ Although human infection is considered rare, the growing number of reports from Thailand,^6^^,^^7^ together with the infection identified in our patient, raises concern that this parasite may represent an emerging zoonosis. This pattern highlights the need for clinical vigilance and suggests a possible shift from isolated spillover events toward localized human transmission.

Human infection with *B* *pahangi* may demonstrate a greater ability to develop within human hosts and produce clinically apparent disease than typically expected for zoonotic filarial nematodes. Although zoonotic species are generally believed to undergo incomplete maturation within human hosts, documented cases of microfilaremia in *B* *pahangi*,^3^ together with findings from volunteer inoculation studies, have shown transient larval circulation.^8^ Over the past 2 decades, accumulating case reports of this parasite have expanded the recognized clinical spectrum, most often describing recurrent or acute lymphangitis without progression to lymphedema, along with extralymphatic involvement such as ocular, breast and subcutaneous lesions.^6^^,^^7^ Notably, several pediatric cases have demonstrated microfilaremia,^6^ supporting that this parasite may be more adaptable in humans than previously appreciated. However, microfilaremia could not be assessed in the present case, as peripheral blood examination was not performed because filariasis was not initially suspected. The patient’s presentation with recurrent lymphangitis and a subcutaneous nodule fits within this expanding spectrum and can mimic other helminthic infections such as gnathostomiasis.^9^ These overlapping features highlight the diagnostic challenge, particularly in areas where both zoonotic filariasis and gnathostomiasis are endemic.

Serologic tests for parasites are generally sensitive but cannot reliably distinguish past exposure from active infection. Cross-reactivity among tissue nematode infections is well recognized, particularly with assays relying on total Ig-based detection, such as immunoblots for *Gnathostoma* antibodies used in this report.^10^ In this case, the positive *Gnathostoma* antibodies may reflect serologic cross-reactivity between zoonotic filariae and *Gnathostoma* species, although prior or subclinical exposure to *Gnathostoma* cannot be excluded. In contrast, filarial IgG4 was negative, a finding consistent with a zoonotic infection rather than a human-specific filarial infection. This ELISA was developed to detect filarial IgG4 using somatic *B. malayi* antigen for the diagnosis of human lymphatic filariasis.^11^ Therefore, it may yield negative results in patients with zoonotic filariasis, as previously reported.^12^ This underscores the limitation of serology in endemic settings and the importance of confirmatory diagnostics. Hence, histopathology and molecular analysis were essential for definitive diagnosis.

Morphologic examination can suggest the taxonomic group and, in some instances, the genus of a nematode, but is frequently limited by parasite degeneration. In this case, the thin, smooth cuticle and the absence of cuticular spines made *Gnathostoma* unlikely, thus definitive identification required molecular analysis. PCR amplification and sequencing of cytochrome c oxidase subunit I and 12S rRNA genes from paraffin-embedded tissue represent reliable methods for species-level identification and were essential for establishing the diagnosis.

There are no standardized treatment guidelines for human *B* *pahangi* infection. Reported zoonotic filariasis cases indicate that surgical excision alone may be sufficient for solitary nodules.^7^ In accordance with WHO guidance, the Thai Ministry of Public Health recommends a 6-day course of diethylcarbamazine (6 mg/kg/d) based on protocols for *B* *malayi* infection.^13^ Although ivermectin effectively eliminates *B* *pahangi* in animals, its efficacy in humans remains uncertain. In our patient, surgical excision combined with 2 doses of oral ivermectin resulted in complete resolution and no recurrence.

This case illustrates a rare human infection with *B* *pahangi* presenting as recurrent lymphangitis and a subcutaneous nodule. Clinical findings closely resembled gnathostomiasis, while serologic results were misleading. Definitive diagnosis required molecular sequencing. Although surgical excision with ivermectin was successful, the lack of species-specific guidelines highlights a therapeutic gap. Recent literature has highlighted the increasing recognition of atypical and zoonotic filarial infections in Thailand.^12^ With rising case numbers and the presence of abundant animal reservoirs and mosquito vectors, *B* *pahangi* should be recognized as an emerging zoonosis warranting increased clinical awareness and improved surveillance.

### Declaration of generative AI and AI-assisted technologies in the writing process

During the preparation of this manuscript, the authors used ChatGPT (OpenAI) to assist with language editing and improving the clarity of the text. After using this tool, the authors critically reviewed and edited the content and take full responsibility for the accuracy and integrity of the published work.

## Conflicts of interest

None disclosed.

## References

[1] World Health Organization (2024). Lymphatic filariasis. https://www.who.int/news-room/fact-sheets/detail/lymphatic-filariasis.

[2] Orihel T.C., Eberhard M.L. (1998). Zoonotic filariasis. Clin Microbiol Rev.

[3] Bhumiratana A., Nunthawarasilp P., Intarapuk A., Pimnon S., Ritthison W. (2023). Emergence of zoonotic *Brugia pahangi* parasite in Thailand. Vet World.

[4] Ravindran R., Varghese S., Nair S.N. (2014). Canine filarial infections in a human Brugia malayi endemic area of India. Biomed Res Int.

[5] Intarapuk A., Bhumiratana A. (2021). Investigation of Armigeres subalbatus, a vector of zoonotic *Brugia pahangi* filariasis in plantation areas in Suratthani, Southern Thailand. One Health.

[6] Suphap N., Somkijrungroj T., Kongwattananon W. (2024). Ocular *Brugia pahangi* filariasis complicated by severe macular damage in Thailand: case report and literature review. Am J Trop Med Hyg.

[7] Thongpiya J., Sa-Nguanraksa D., Samarnthai N., Sarasombath P.T. (2021). Filariasis of the breast caused by *Brugia pahangi*: a concomitant finding with invasive ductal carcinoma. Parasitol Int.

[8] Edeson J.F., Wilson T., Wharton R.H., Laing A.B. (1960). Experimental transmission of *Brugia malayi* and *B. pahangi* to man. Trans R Soc Trop Med Hyg.

[9] Lupi O., Downing C., Lee M. (2015). Mucocutaneous manifestations of helminth infections: nematodes. J Am Acad Dermatol.

[10] Ieamsuwan I., Watthanakulpanich D., Chaisri U., Adisakwattana P., Dekumyoy P. (2021). Evaluation of immunodiagnostic tests for human gnathostomiasis using different antigen preparations of Gnathostoma spinigerum larvae against IgE, IgM, IgG, IgG1-4 and IgG1 patterns of post-treated patients. Trop Med Int Health.

[11] Wongkamchai S., Satimai W., Loymek S., Nochot H., Boitano J.J. (2015). An ELISA kit with two detection modes for the diagnosis of lymphatic filariasis. J Helminthol.

[12] Sarasombath P.T., Sitthinamsuwan P., Wijit S. (2024). Integrated histological and molecular analysis of filarial species and associated wolbachia endosymbionts in human filariasis cases presenting atypically in Thailand. Am J Trop Med Hyg.

[13] Sakaraseni O., Krobtrakulchai T., Mothana N., Jiraphongsa C. (2017). Filariasis Brugia pahangi prevention and control guideline. Wkly Epidemiol Surveill Rep Thai.

